# An immunoassay for the measurement of (1→3)-β-D-glucans in the indoor environment

**DOI:** 10.1080/09629359791578

**Published:** 1997-11

**Authors:** Jeroen Douwes, Gert Doekes, Roy Montijn, Dick Heederik, Bert Brunekreef

**Affiliations:** 1 Environmental and Occupational Health Department of Environmental Sciences Wageningen Agricultural University PO Box 238 Wageningen 6700 AE The Netherlands; 2 Institute of Molecular Cell Biology BioCentrum Amsterdam University of Amsterdam The Netherlands

## Abstract

An inhibition enzyme immunoassay was developed for quantitation of (1→3)-β-D-glucans in the indoor environment. Immunospecific rabbit antibodies were produced by immunization with bovine serum albuminconjugated laminarin.The laminarin calibration curve ranged from 40 to 3000 ng/ml.Another (1→3)-β-D-glucan (curdlan) showed a similar inhibition curve, but was less reactive on a weight basis. Pustulan, presumed to be (1→3)-β-D-glucan, also showed immunoreactivity in the assay. Control experiments indicated that this was due to (1→3)-β-D-glucan structures. Other non-(1→3)-β-D-glucan polysaccharides did not react. (1→3)-β-Dglucan was detectable in dust from a variety of occupational and environmental settings. We conclude that the new assay offers a useful method for indoor (1→3)-β-Dglucan exposure assessment.


Mediators of Inflammation, **6,** 257–262 (1997)

### An immunoassay for the measurement of (1→3)-β-D-glucans in the indoor environment

**Jeroen Douwes[1,CA], Gert Doekes[1], Roy Montijn[2], Dick Heederik[1] and Bert Brunekreef[1]**

[1]Environmental and Occupational Health, Department of Environmental Sciences, Wageningen Agricultural University, PO Box 238, 6700 AE Wageningen, the Netherlands, and [2]Institute of Molecular Cell Biology, BioCentrum Amsterdam, University of Amsterdam, the Netherlands

[CA]Corresponding author
Tel: +31 7 482080/482595
Fax: +31 7 485278
Email: jeroen.douwes@staff.eoh.wau.nl

**An inhibition enzyme immunoassay was developed for quantitation of (1→3)-β-D-glucans in the indoor environment. Immunospecific rabbit antibodies were produced by immunization with bovine serum albumin-conjugated laminarin. The laminarin calibration curve ranged from 40 to 3000 ng/ml. Another (1→3)-β-D-glucan (curdlan) showed a similar inhibition curve, but was less reactive on a weight basis. Pustulan, presumed to be (1→6)-β-D-glucan, also showed immunoreactivity in the assay. Control experiments indicated that this was due to (1→3)-β-D-glucan structures. Other non-(1→3)-β-D-glucan polysaccharides did not react. (1→3)-β-D-glucan was detectable in dust from a variety of occupational and environmental settings. We conclude that the new assay offers a useful method for indoor (1→3)-β-D-glucan exposure assessment.**

**Keywords:** Mold (1→3)-β-D-glucan, organic dust, occupational and home environment, immunoassay

### Introduction

Exposure to bio-aerosols in indoor environments can induce toxic, inflammatory and allergic reactions, resulting in respiratory symptoms. It has been suggested recently that (1→3)-β-D-glucans contribute to bio-aerosol-induced inflammatory responses and the resulting respiratory symptoms and complaints [1–4]. (1→3)-β-D-glucans, which may originate from a large variety of sources including molds, some bacteria and most plants, can initiate a variety of immunobiological responses in vertebrates [5–12].

At present, knowledge about airborne (1→3)-β-D-glucan exposure as a potential respiratory health hazard is limited owing to a lack of generally available methods to measure environmental (1→3)-β-D-glucan in field studies.

There is a clear need for an improved, specific, sensitive and cost-efficient method to quantify (1→3)-β-D-glucans that can be used in large-scale environmental hygiene and epidemiological studies. Here, we describe a new assay that meets these requirements. The new assay was applied on organic dust samples from various environments, various plant materials and the culture fluid of yeast cultures.

### Materials and methods

### Glucans

The following glucans and related saccharides were used in this study: laminarin, β(1→3)(1→6)-glucan [13,14]; CM-curdlan, (1→3)-β-D-glucan [13,14]; pustulan, (1→6)-β-D-glucan [23]; β(1→6)(1→3)-glucan [15]; dextran, (1→6)(1→3)(1→4)(1→2)-glucan [13]; and mannan, (1→2)(1→3)(1→6)-mannan. Laminarin and pustulan were dissolved in $H_2O$ at 120°C, curdlan in 0.05 mol/l NaOH and dextran and mannan in $H_2O$.

### Bovine serum albumin–laminarin conjugate for immunization

(1→3)-β-D-glucan (laminarin) was coupled to bovine serum albumin for immunization purposes. The immunoreactivity of (1→6)-β-D-glucan structures present in laminarin was abolished by oxidation with 0.25 mol/l $NaIO_4$. Conjugation of oxidized laminarin to bovine serum albumin was performed by reductive amination [16]. The glucan–bovine serum albumin conjugate contained approximately 8% (weight/weight) carbohydrate.

© 1997 Rapid Science Publishers

### Rabbit anti-(1→3)-β-glucan antibodies

Rabbits were immunized by subcutaneous injection of 1 ml glucan conjugate solution (0.1 mg protein/ml). Specific antibodies were isolated from ammonium sulphate-precipitated immunoglobulins by affinity chromatography using epoxy-activated Sepharose on which (1→3)-β-D-glucan was coupled, as described by Hutchins and Bussey [17].

### Inhibition enzyme immunoassay

Laminarin (2 μg/ml) in phosphate-buffered saline (pH 7.0) was coated overnight onto each well (200 μl/well) of a microtiter plate at 4°C. After washing, unreacted sites were blocked with 0.5% gelatine. A test sample (four dilutions) or laminarin standard (12 dilutions; 9.8 ng/ml to 20 μg/ml) was added (100 μl) in a microwell and subsequently mixed with an equal volume of affinity-purified anti-(1→3)-β-D-glucan antibodies diluted 1:75 000 and incubated for 1.5 h at 37°C. After washing, 200 μl peroxidase-labeled horse antirabbit immunoglobulin antibodies (1:5000) was added and incubated for 1 h at 37°C. After washing, 200 ml *o*-phenylenediamine (2 mg/ml) containing 0.015% $H_2O_2$ was added and incubated for 30 min at 20°C. The reaction was terminated with 50 μl 2 N HCl, and the optical density was read at 492 nm. (1→3)-β-D-glucan concentrations were calculated using a four-parameter curve-fitting program.

### (1→3)-β-glucanase and $NaIO_4$ treatment

Specific destruction of (1→3)-β-D-glucan or (1→6)-β-D-glucan conformational structures was accomplished by treatment with specific (1→3)-β-glucanase (zymolyase 100T) [18] or 0.25 mol/l $NaIO_4$ [19], respectively. Specific anti-(1→6)-β-D-glucan rabbit antibodies were used to confirm the efficacy of $NaIO_4$ treatment and the specificity of the (1→3)-β-glucanase activity of zymolyase. Anti-(1→6)-β-D-glucan antibodies were raised in rabbits as described by Montijn *et al.* [19].

### Plant samples

Extracts were made of cereals (wheat, barley and corn flour), soybeans, tapioca and potato using two extraction procedures: heat and alkaline treatment. Each product was suspended (1% weight/volume) and homogenized in bidistilled water with 0.05% Tween-20 and in 0.05 mol/l NaOH. The samples suspended in bidistilled water with 0.05% Tween-20 were autoclaved at 120°C (1 bar) for 1 h. The samples suspended in 0.05 mol/l NaOH were rocked vigorously for 2 h. Sample suspensions were centrifuged at 1000 **g** for 15 min and the supernatants stored at –20°C.

### Yeast samples

Cell-free yeast culture media were tested, in which wild-type and various mutants of *Saccharomyces cerevisiae* strain FY384 had been cultured [20]. Yeasts were grown at 28°C to the early exponential phase in standard minimal medium. Culture medium in which no yeasts had been cultured was used as a control.

### Environmental dust samples

Inhalable airborne dust-sampling, both area and personal, was carried out in two waste-composting facilities, and personal inhalable dust samples were collected from pig farmers. The inhalable dust was collected on glass-fiber filters using Personal Air Samplers sampling heads (PAS-6; Agricultural University, Wageningen, the Netherlands) at a flow rate of 2 liters/min [21]. Settled dust samples from the floors of living rooms, bedrooms and kitchens and from mattresses were collected in a series of 25 German homes on paper filters according to an internationally standardized protocol [22].

Airborne dust samples were extracted in 5 ml and settled dust samples in 5–20 ml pyrogen-free water with 0.05% Tween-20 (≈10% weight/volume). The extraction procedure applied was the same as described for heat extraction of plant samples. After extraction, the samples were centrifuged at 1000 **g** for 15 min, and the supernatant was stored at –20°C.

### Results

### Specificity of the inhibition enzyme immunoassay

Fig. 1 gives dose–response curves for two (1→3)-β-D-glucan preparations and various other polysaccharides. The inhibition curve for laminarin ranged from approximately 40 to 3000 ng/ml (15–85% inhibition). The other (1→3)-β-D-glucan (curdlan) showed a parallel inhibition curve but was three to five times less reactive on a weight basis. Of the other polysaccharides tested, only pustulan, presumed to contain exclusively (1→6)-β-D-glucan [23], showed a parallel dose–response curve at concentrations approximately ten times higher than laminarin. The other polysaccharides were all incapable of inhibiting the anti-(1→3)-β-D-glucan antibodies.

Control experiments with $NaIO_4$ and (1→3)-β-D-glucanase treatment were performed to determine whether the inhibitory activity of pustulan was a result of the presence of β(1→3)-glucosidic structures in that preparation. Zymolyase ((1→3)-β-D-glucanase) almost completely abolished the inhibitory activity of both glucans (Fig. 2a), whereas periodate treatment did not abolish the inhibitory capacity of the preparations (Fig. 2b). Additional control experi-

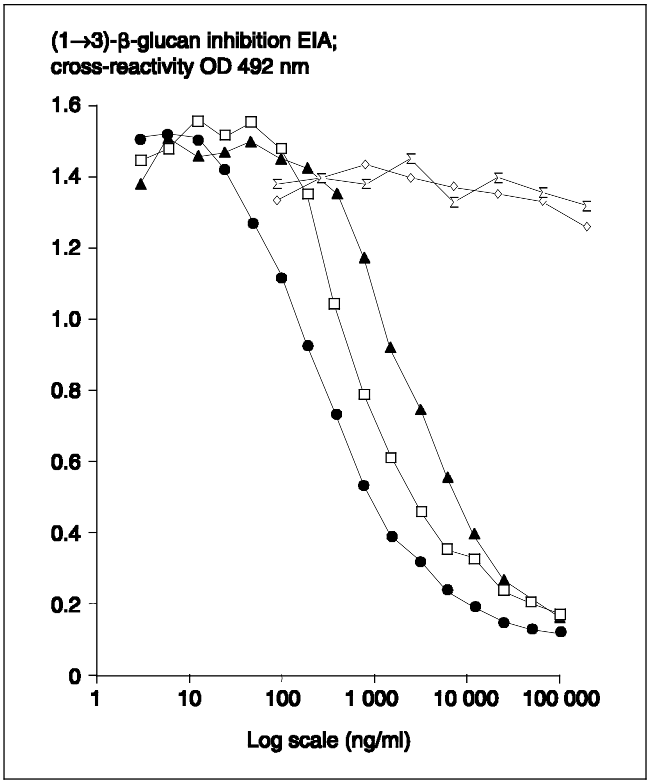

FIG. 1. Inhibition–dilution curves of four different glucans and mannan. Laminarin (closed circles) and CM-curdlan (open squares) are both (1→3)-β-glucans, pustulan (closed triangles) is a (1→6)-β-D-glucan contaminated with (1→3)-β-glucan, dextran (open diamonds) is an α(1→6)-glucan and mannan (crosses) is polymannose. EIA, enzyme immunoassay; OD, optical density.

ments with (1→6)-β-D-glucan inhibition enzyme immunoassays confirmed that the periodate treatment abolished the immunoreactivity of (1→6)-β-D-glucan structures, whereas zymolyase had no effect (Fig. 2c and d).

Thus, the cross-reactivity observed with pustulan in the (1→3)-β-D-glucan enzyme immunoassay was due to the presence of (1→3)-β-D-glucan structures in the preparation, and not to a lack of specificity of the anti-(1→3)-β-D-glucan antibodies.

### Sensitivity and reproducibility of the inhibition enzyme immunoassay

The detection limit was set at 15% inhibition, which corresponded to 40 ng/ml. The resulting detection limits for airborne and settled dust were 200 ng/$m^3$ and 0.5 µg/g dust, respectively.

Dilution curves obtained with autoclaved (120°C) extracts of the environmental samples were essentially parallel to the calibration curve. The reproducibility of the inhibition enzyme immunoassay was determined on detectable duplicate analyses of the environmental dust extracts and expressed as the coefficient of variation. Mean coefficients of variation for the inhibition assay of 20% ($n$ = 85) and 27% ($n$ = 100) were calculated on the basis of the occupational samples and the house dust samples, respectively.

### (1→3)-β-D-glucan in plant extracts

All plant extracts tested contained measurable amounts of (1→3)-β-D-glucan, and concentrations ranged from 0.01% (w/w) for potato to 0.7% (w/w) for barley. Differences between the results obtained with two extraction methods (heat versus alkaline extraction) were small and not systematic.

### (1→3)-β-D-glucan in yeast culture media

The (1→3)-β-D-glucan levels in yeast culture media were moderate, ranging between approximately 0.2 and 1 µg/ml, while no immunoreactivity was detected in the medium without yeast.

### Exposure levels in various environments

Appreciable levels of (1→3)-β-D-glucan were measured in two occupational environments, previously characterized as high bio-aerosol exposure environments (Table 1) [23,24]. House dust also contained substantial amounts of (1→3)-β-D-glucan (Table 2). (1→3)-β-D-glucan levels are expressed as geometric means with geometric standard deviations, as the exposure data in these environments are generally best described by a log-normal distribution. Concentrations below the detection limit were considered to have a value of two-thirds of this limit [25].

Blank glass fiber filters did not contain detectable levels of (1→3)-β-D-glucan. However, heat extracts of blank paper filters used for house-dust sampling contained significant amounts of (1→3)-β-D-glucans (188 µg, SD 39.3, $n$ = 6). After correction, (1→3)-β-D-glucans remained detectable in all house-dust samples (Table 2).

### Discussion

Two different linear (1→3)-β-D-glucans (curdlan and laminarin) reacted in the inhibition assay. The antibodies also reacted with pustulan, which is presumed to contain only β(1→6)-glucan [13]. However, the reactivity of pustulan, like that of laminarin, was retained after $NaIO_4$ treatment, whereas it was completely lost after treatment with (1→3)-β-glucanase. Similar experiments with a polyclonal anti-(1→6)-β-D-glucan antiserum confirmed the specific (1→3)-β-glucanase activity of zymolyase and the complete destruction of (1→6)-β-D-glucan structures by $NaIO_4$ treatment. Consequently, we conclude that pustulan

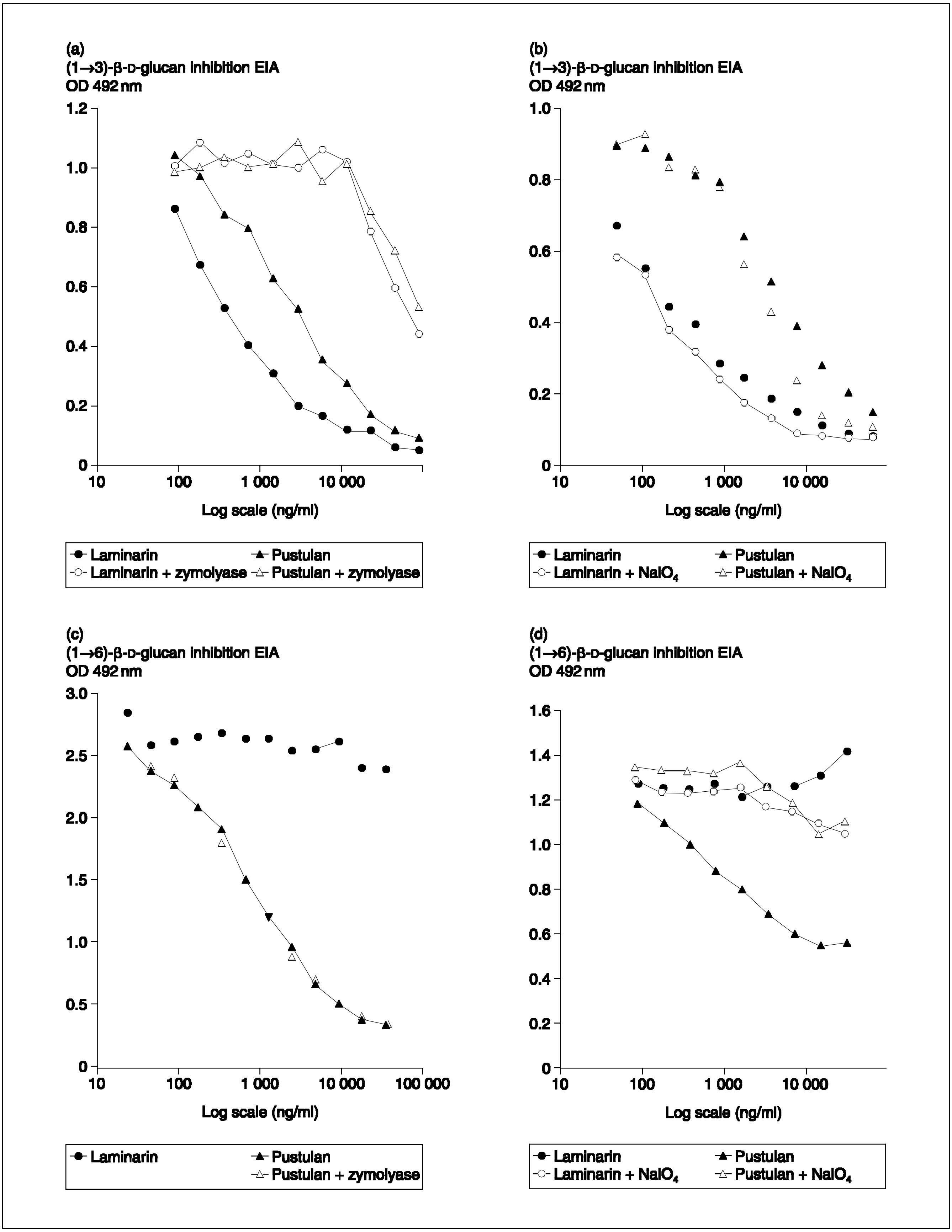

FIG. 2. Inhibition–dilution curves for untreated and zymolyase-treated laminarin and pustulan in (a) (1→3)-β-D-glucan enzyme immunoassay (EIA) and (c) (1→6)-β-D-glucan EIA, and for untreated and $NaIO_4$-treated laminarin and pustulan in (b) (1→3)-β-D-glucan EIA and (d) (1→6)-β-D-glucan EIA. OD, optical density.

**Table 1.** (1→3)-β-D-glucan exposure measurements in two occupational environments (glucan detectable/total samples)

| Sampling location | GM (μg/m³) | GSD | Minimum | Maximum |
|---|---|---|---|---|
| **Waste composting facility** | | | | |
| Plant 1 | | | | |
| Offices, control rooms (0/2) | <0.2 | – | – | – |
| Compost ripening (4/4) | 1.02 | 2.1 | 0.53 | 2.95 |
| Process hall (12/12) | 19.35 | 1.6 | 11.00 | 47.03 |
| Plant 2 | | | | |
| Offices, control rooms (0/5) | <0.2 | – | – | – |
| Compost ripening (1/6) | 0.39 | 11.0 | <0.2 | 28.61 |
| Process hall (2/16) | <0.2 | 1.6 | <0.2 | 0.51 |
| Unloading organic waste (8/8) | 3.80 | 2.1 | 2.02 | 15.97 |
| Personal (19/21) | 6.57 | 7.2 | <0.2 | 210.11 |
| **Swine confinement workers** | | | | |
| Personal (55/59) | 4.34 | 3.4 | <0.2 | 38.49 |

GM, geometric means, calculated to include non-detectable results with non-detectable values set at two-thirds of the detection limit (0.2μg/m³) [24]; GSD, geometric standard deviations. Ambient air was sampled except where indicated as personal air.

**Table 2.** (1→3)-β-glucan exposure measurements in house dust (glucan detectable/total samples)

| Sampling location | GM (μg/g) | GSD | Minimum | Maximum |
|---|---|---|---|---|
| Living room (25/25) | 1293 | 1.4 | 627 | 2915 |
| Bedroom (25/25) | 1286 | 1.7 | 408 | 3507 |
| Kitchen (25/25) | 1168 | 2.0 | 376 | 6540 |
| Mattress (25/25) | 757 | 1.7 | 182 | 1654 |

GM, geometric means; GSD, geometric standard deviations.

also contains (1→3)-β-glucan structures. Since all other polysaccharides tested were not reactive in our assay, we conclude that the inhibition enzyme immunoassay specifically recognizes (1→3)-β-D-glucan epitopes. Preliminary results indicating reactivity with yeast and plant glucans (see below), mainly consisting of β(1→3)(1→6) and β(1→3)(1→4) linkages, respectively, suggest that both linear and branched (1→3)-β-glucans are recognized in the inhibition assay.

Another method for measuring (1→3)-β-D-glucans with the use of a glucan-reactive preparation of *Limulus* amebocyte lysate has recently been described [3,26]. This test is very sensitive (1–10 pg/ml) but probably not highly specific, since it also reacts with other glucans, and with very high concentrations of other polysaccharides [14,27].

The use of a specific immunoassay that is also less expensive may be advantageous in hygiene and epidemiological-effect studies.

Thus far, discussions on the immunobiological effects of glucans have mainly focused on fungal glucans. It is not clear whether plant glucans have similar properties. Plant glucans such as laminarin and barley β-D-glucan also interact with the *Limulus* coagulation factor G [14]. This suggests that plant glucans are also biologically active, and the detection of both plant and fungal (1→3)-β-glucans in a (1→3)-β-glucan assay (described in this paper) may therefore be relevant.

Very high (1→3)-β-D-glucan levels were found in the occupational and residential dust samples studied. Comparable concentrations (1 μg/m³) have been measured in an experimental cotton cardroom using a glucan-reactive *Limulus* amebocyte lysate assay [28]. The high (1→3)-β-glucan content in organic dust of both occupational and residential origins (0.5–20%) suggests a large proportion of plant and/or fungal material in that dust. This seems plausible considering that (1→3)-β-glucans occur as major structural cell-wall or storage components of many plants and microorganisms, which are known to contribute largely to the content of most organic dust. Moreover, (1→3)-β-D-glucans are likely to be relatively degradation-resistant in the environment, resulting in a high carbohydrate content in organic dust.

In conclusion, the (1→3)-β-D-glucan inhibition assay described here offers a specific and sensitive method for indoor (1→3)-β-D-glucan exposure assessment. The new assay is therefore expected to be useful in epidemiological studies to investigate the relationship between (1→3)-β-D-glucan exposure and respiratory health.

### References

1. De Lucca AJ II, Brogden KA, French AD: Agglutination of lung surfactant with glucan. *Br J Ind Med* 1992, **49**:755–760.
2. Fogelmark B, Sjöstrand M, Rylander R: Pulmonary inflammation induced by repeated inhalations of β(1→3)-D-glucan and endotoxin. *Int J Exp Pathol* 1994, **75**:85–90.
3. Rylander R, Persson K, Goto H, Yuasa K, Tanaka S: Airborne β-1,3-glucan may be related to symptoms in sick buildings. *Indoor Environ* 1992, **1**:263–267.
4. Williams DL: (1→3)-β-D-glucans. In: *Organic Dusts: Exposure, Effects, and Prevention*. Edited by Rylander R, Jacobs RR. Chicago (IL): Lewis Publishers; 1994:83–85.
5. Adachi Y, Okazaki M, Ohno N, Yadomae T: Enhancement of cytokine production by macrophages stimulated with (1→3)-β-D-glucan, grifolan (GRN), isolated from *Grifola frondosa*. *Biol Pharm Bull* 1994, **17**:1554–1560.
6. Brattgjerd S, Evensen , Lauve A: Effect of injected yeast glucan on the activity of macrophages in Atlantic salmon, *Salmo salar* L., as evaluated by *in vitro* hydrogen peroxide production and phagocytic capacity. *Immunology* 1994, **83**:288–294.
7. Di Luzio NR: Lysozyme, glucan activated macrophages and neoplasia. *J Reticuloendothelial Soc* 1979, **26**:67–81.
8. Kraus J, Franz G: β(1→3) glucans: anti-tumor activity and immunostimulation. In: *Fungal Cell Wall and Immune Response*. Edited by Latgé JP, Boucias D. Berlin: Nato ASI series H53, Springer-Verlag; 1991:431–444.
9. Ramani M, Howell CJ, Spur BW, *et al.*: The generation and cellular distribution of leukotriene C4 in human eosinophils stimulated by unopsonized zymosan and glucan particles. *Clin Immunol* 1988, **81**:696–705.
10. Saito K, Nishijma M, Ohno N, Nagi N, Yadomae T, Miyazaki T: Activation of complement and *Limulus* coagulation system by an alkali-soluble glucan isolated from *Omphalia lapidescens* and its less branched derivates. *Chem Pharm Bull* 1992, **40**:1227–1230.
11. Sakurai T, Ohno N, Yadomae T: Changes in immune mediators in mouse lung produced by administration of soluble (1→3)-β-D-glucan. *Biol Pharm Bull* 1994, **17**:617–622.
12. Zhang K, Petty HR: Influence of polysaccharides on neutrophil function: specific antagonists suggest a model for cooperative saccharide-associated inhibition of immune complex-triggered superoxide production. *J Cell Biochem* 1994, **56**:225–235.
13. Stone BA, Clarke AE: *Chemistry and Biology of (1→3)-β-glucans*. Melbourne, Australia: La Trobe University Press; 1992.
14. Tamura H, Arimoto Y, Tanaka S, Yoshida S, Obayashi T, Kawai T: Automated kinetic assay for endotoxin and (1→3)-β-D-glucan in human blood. *Clin Chim Acta* 1994, **226**:109–112.
15. Hirata A, Adachi Y, Itoh W, Komoda M, Tabata K, Sugawara I: Monoclonal antibody to proteoglycan derived from *Grifola frondosa* (Maitake). *Biol Pharm Bull* 1994, **17**:539–524.
16. Roy R, Katzenellenbogen E, Jennings HJ: Improved procedures for the conjugation of oligosaccharides to protein by reductive amination. *Can J Biochem Cell Biol* 1984, **62**:270–275.
17. Hutchins K, Bussey H: Cell wall receptor for yeast killer toxin: involvement of (1→6)-β-D-glucan. *J Bacteriol* 1983, **154**:161–169.
18. Tanaka S, Aketagawa J, Takahashi S, Shibata Y, Tsumuraya Y, Hashimoto Y: Activation of a limulus coagulation factor G by (1→3)-β-D-glucans. *Carbohydr Res* 1991, **218**:167–174.
19. Montijn RC, van Rinsum J, van Schagen A, Klis F: Glucomannoproteins in the cell wall of *Saccharomyces cerivisiae* contain a novel type of carbohydrate side chain. *J Biol Chem* 1994, **269**:19338–19342.
20. Winston L, Dollard C, Ricupero-Havasse SL: Construction of a set of convenient *Saccharomyces cerevisiae* strains that are isogenic to S288C. *Yeast* 1995, **11**:53–55.
21. Ter Kuile WM: Comparison of various sampling devices for the assessment of total dust concentrations in the workplace [in German]. *Staub Reinh Luft* 1984, **44**:211–216.
22. Platts-Mills TAE, De Weck AL: Dust mite allergens and asthma: a world wide problem. International workshop report. *J Allergy Clin Immunol* 1989, **11**:199–206.
23. Preller L, Heederik D, Kromhout H, Boleij JSM, Tielen JMT: Determinants of dust and endotoxin exposure of pig farmers: development of a control strategy using empirical modelling. *Ann Occup Hyg* 1995, **39**:545–557.
24. Clark CS, Rylander R, Larsson L: Levels of Gram-negative bacteria, *Aspergillus fumigatus*, dust and endotoxin at compost plants. *Appl Environ Microbiol* 1983, **5**:1501–1505.
25. Helsel DR: Less than obvious: statistical treatment of data below the detection limit. *Environ Sci Technol* 1990, **24**:1766–1774.
26. Obayashi T, Yoshida M, Mori T, *et al.*: Plasma (1→3)-β-D-glucan measurement in diagnosis of invasive deep mycosis and fungal febrile episodes. *Lancet* 1995, **345**:17–20.
27. Mikami T, Nagase T, Matsumoto T, Suzuki S, Suzuki M: Gelation of *Limulus* Amoebocyte lysate by simple polysaccharides. *Microbiol Immunol* 1982, **26**:403–409.
28. Rylander R, Bergström R, Goto H, Yuasa K, Tanaka S: Studies on endotoxin and β(1→3)-D-glucan in cotton dust. In: *Proceedings of the Thirteenth Special Session on Cotton Dust Research*. Edited by Jacobs RR, Wakelyn PJ. Memphis, Tennessee: National Cotton Council; 1989:46–47.
